# Personality traits among the staff of moroccan call centers

**DOI:** 10.1192/j.eurpsy.2021.1191

**Published:** 2021-08-13

**Authors:** E. Drissi, S. Boulbaroud, H. Hami, A. Ahami, F.Z. Azzaoui

**Affiliations:** 1 Laboratory Of Biology And Health, Faculty of Science, Ibn Tofail University, Kenitra, Morocco; 2 Laboratory Of Biotechnology And Sustainable Development Of Natural Ressources, Polydisciplinary Faculty, Sultan Moulay Slimane University, Benimellal, Morocco; 3 Laboratory Of Natural Ressources And Sustainable Development, Faculty of Science, Ibn Tofail University, Kenitra, Morocco

**Keywords:** Personality, Call centers, employees, morocco

## Abstract

**Introduction:**

The aim of this research is to study the five major personality traits in a sample of call center employees in the Rabat-Salé-Kénitra Region, Morocco.

**Objectives:**

Demonstrate the domination of certain personality dimensions over others in this population.

**Methods:**

This is a cross-sectional epidemiological study that involved 121 individuals, including 59 men and 62 women with an average age of 31.74 ± 7.93. The personality traits were assessed using the Big Five test.

**Results:**

The results show that, (63.64%) of our sample has a more dominant extraversion pole, while (36.36%) of the participants have a more dominant introversion pole. (66.12%) of our study subjects have a dominance of agreeableness dimension, while for (33.88%) of the sample the disagreeableness dimension dominates. Almost parity is observed between those in our sample who have a dominance of the conscientiousness pole (50.41%), and those who have a dominance of the impulsivity pole (49.59%). 74.38% of the participants have a dominance of the neuroticism pole, while (25.62%) have a dominance of the emotional stability pole. (57.02%) has a dominance of the openness pole, while (42.98%) have a dominance of the closedness pole.

**Conclusions:**

This study sample is characterized to a large extent by extraversion and agreeableness; moreover this study has shed light on the dominance of the neuroticism trait in this kind of population. However, considering this study concerned only one region, it would be interesting to widen the geography of the survey to acquire more exhaustive results.

